# Response of Ecosystem Health to Land Use Changes and Landscape Patterns in the Karst Mountainous Regions of Southwest China

**DOI:** 10.3390/ijerph19063273

**Published:** 2022-03-10

**Authors:** Zhijie Wang, Yan Liu, Yixin Li, Yuan Su

**Affiliations:** 1 College of Life Sciences, Guizhou University, Guiyang 550025, China; zjwang3@gzu.edu.cn; 2 Collaborative Innovation Center for Mountain Ecology & Agro-Bioengineering, Guiyang 550025, China; 3 College of Forestry, Guizhou University, Guiyang 550025, China; liuyan09022022@163.com (Y.L.); yixin773145@163.com (Y.L.)

**Keywords:** ecosystem health, vigor–organization–resilience framework, land use change, landscape composition and configuration, karst mountainous region

## Abstract

The quantitative assessment of ecosystem health is important for interpreting the ecological effects of land use changes and formulating effective measures of sustainable ecological development by policymakers. This study investigated the response of ecosystem health to land use changes and landscape patterns in the karst mountainous regions of southwest China by taking Guiyang City as a case study area and assessing the spatial and temporal changes in ecosystem health from 2008 to 2017 using the vigor–organization–resilience model; it analyzed the influence of land use changes and landscape patterns on ecosystem health using spatial overlay analysis, the Dunnett’s T3 test, and the Spearman correlation analysis. The results show that the land use structure dramatically changed, with a trend of a sharp decrement of farmland and rapid increment of forestland and construction land due to rapid urbanization and ecologization. The overall ecosystem health was at a relatively strong level, with the average value greater than 0.6. The deterioration of ecosystem health was attributed to the expansion of construction land and farmland and the degradation of forestland, while the increment of forestland was the major contributor to the improvement of ecosystem health. The ecosystem health of the forestland + farmland landscape was significantly superior to that of forestland + construction land and construction land + farmland landscapes. Moreover, each landscape configurations had a significant positive or negative correlation with the ecosystem health. This study provides a valuable reference for formulating sustainable environmental management strategies in karst mountainous regions in China.

## 1. Introduction

Natural ecosystems provide both the material basis and ecological services for human survival and development, and a healthy ecosystem is a fundamental guarantee of sustainable social and economic development [1,2,3]. However, due to the expanded breadth and intensity of human activities, rapid urbanization and industrialization have led to unprecedented changes to the global ecosystem, resulting in the degradation of the ecosystem and its services, which poses a serious threat to the survival and sustainable development of human society [1,4,5,6]. Ecosystem health is the purpose and basis of environmental management, and the core of comprehensive ecosystem evaluation; thus, increasing attention has been paid to assessing ecosystem health and the impact of human activities on ecological health in recent decades [7,8,9,10,11].

The concept of ecological health was first proposed in [12], which enriched the study of ecosystems and has gained wide acceptance among scientists [2,13]. Scholars have since supplemented and improved the concept of ecosystem health [4,10,13,14,15,16]. In general, ecosystem health refers to the self-organization, self-maintenance, and recovery ability of natural ecosystems under conditions of stress [4,12], and the ability to meet the reasonable requirements of human society [1,15]; it reflects the ecosystem’s stability and sustainability [12]. In addition, ecosystem health represents the regional ecological quality from two aspects of ecosystem structure and function and is an important basis for comprehensive ecosystem evaluation and management at the macro scale [1,11]. Costanza [13] pointed out that a healthy ecosystem is based on the three main aspects of vigor, organization, and resilience, which provide an effective scientific research perspective and method for assessment [17]. Therefore, in recent years, numerous methods and models for assessing ecosystem health have been developed, such as the pressure–state–response framework [17,18,19,20], the driving force–pressure–state–impact–response–management framework [21], and the natural–social–economic model [22]. The vigor–organization–resilience (VOR) paradigm, the most widely accepted classical framework, is still widely used in relevant studies [2,7,11,23].

Land use change not only has caused tremendous changes to the surface landscape structure, but also has affected materials cycles and energy flows of landscapes, profoundly impacting regional ecological processes [1,24,25,26]. Rapport [4] pointed out that a healthy ecosystem has an integral structure and is resistant to external disturbances, whilst also offering sustainable resources for humans. The structural and functional integrity of a landscape are basic prerequisites for maintaining ecosystem health and achieving sustainable development [27]. Therefore, regional ecological health assessment based on landscape ecology theory is considered an effective method to research the impact of spatial patterns on ecological processes at a regional scale [7].

A quantitative study of the response of ecological health to regional land use change could be conducive to a comprehensive evaluation of the macroecological effects of land use change [1]. Several scholars have studied the effects of land use/land cover change and its related transformation of landscape patterns with regard to ecological health [1,2,9,20]. However, the relevant research perspective was mainly focused on the ecological health response under the single effect of land use change in rapidly urbanizing, rural, or ecological restoration regions [7,11,18,19,22,27,28]. In fact, with the rapid development of social economy and the continuous strengthening of human efforts in ecological protection, the land use landscape of some typical regions displays the coupling of ecologization and urbanization with a more complication change in land use structure, and the impact of land use change on ecological health is also more complex [10]. On the other hand, there are few studies on the response of ecological health to land use change under the coupling of ecologization and urbanization. Thus, it is vital to scientifically monitor and assess the temporal and spatial changes in regional ecological health under the coupling of ecologization and urbanization in order to formulate regional sustainable development policies now and in the future. In addition, research on the influence of spatial patterns on ecological health and their relationship from the landscape perspective has attracted attention in recent years [1,2]. Peng et al. [2] pointed out that a simple analysis of ecosystem structure in terms of regional ecosystem health at the landscape scale should be incomplete because the landscape composition and configuration are being substantially changed by humankind, affecting the stability of landscape structure and ecosystem services [15,29]. Therefore, understanding the response of regional ecosystem health from the perspective of landscape structure is an urgent scientific issue with regard to regional ecological health assessment and environmental management.

The southwest karst mountainous region of China, represented by Guiyang City, Guizhou Province, is characterized by abundant natural ecosystem resources with rich biodiversity and a fragmented heterogeneous landscape [30,31]. The plant biodiversity in this region comprises 30–40% southern China flora, and there are many nationally and internationally protected plant and animal species. For example, 123 species (31.6% of the total) appeared on the first iteration of the list of nationally protected vegetation species [32]. As this region has a unique geological setting and high landscape heterogeneity, the eco-environmental sensitivity is so high that once a strong artificial disturbance occurs, it is difficult to rehabilitate the environment and rebuild the ecosystem [33,34]. For example, in recent years, under the multiple influences of social economic development, the urbanization process has been accelerating, which has worsened the effects on the fragile environment and caused an intensification of landscape fragmentation, leading to the declined EH in urban expansion areas [35,36,37]. Meanwhile, ecological protection and restoration measures have been largely implemented, and the overall environmental quality has obviously improved [37]. At present, the coupled development of land use patterns driven by ecologization and urbanization is typical of land use landscape with the trend of forestland and construction land synchronously increasing, resulting in a complex impact on ecosystem health [37,38]. This provides an ideal study area to perform a regional ecosystem health assessment under the coupled effect of urbanization and ecologization. Moreover, the heterogeneous karst landscape and diverse land use types provide abundant research objects to revealing the relationship between regional ecological health and landscape patterns.

Taking Guiyang City as a case study, a spatial and temporal assessment of ecological health was conducted by adopting the most widely used VOR framework. The aims of this study were as follows: (1) to analyze the spatial and temporal changes in land use patterns under the coupled influence of urbanization and ecological restoration; (2) to determine the response of ecological health to different types of land use change; (3) to explore the differences in ecological health in different landscape structures and compositions, and the relationship between landscape configuration and ecological health.

## 2. Materials and Methods

### 2.1. Study Area

**Figure 1:**
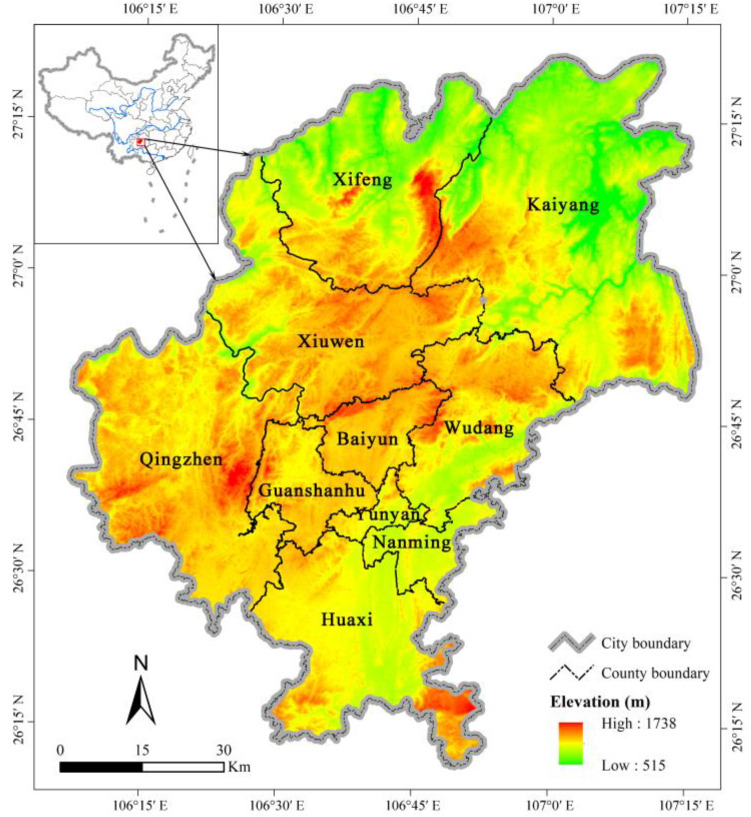
Location of Guiyang City.

Guiyang City (106°07′–107°17′ E, 26°11′–27°22′ N), a typical karst mountainous region in southwest China, is located in a hilly area of the middle mountains of Guizhou Province, and the watershed between the Yangtze and Pearl Rivers. Guiyang landform consists of mountains and hills (Figure 1). The karst environment is fragile, and the climate type in the region is a subtropical monsoon climate with an average annual temperature and precipitation of 15.3 °C and 1197–1248 mm, respectively [39]. As the capital of Guizhou Province, Guiyang City experienced rapid urbanization in the last decades [38]. The Chinese government implemented a series of ongoing ecological restoration projects in the study area to restore the natural conditions of the ecosystem [18]. The study area is 1 of the richest biodiversity regions in China, with diverse vegetation types; the average vegetation coverage reached 50% in 2020. The primary natural vegetation type in the region is subtropical evergreen forests. The dominant canopy species in natural forest stands are in the Fagaceae and Lauraceae families, while plants from Theaceae and Magnoliaceae families are also common [36]. The main vegetation types of artificial and secondary forest are evergreen broad-leaved, evergreen coniferous, and mixed coniferous and broad-leaved forests, and the main tree species are *Pinus massoniana*, *Pinus armandii*, *Ligustrum lucidum*, *Cryptomeria japonica*, *Cunninghamia lanceolate*, *Cinnamomum longepaniculatum*, and *Betula alnoides*, etc. Therefore, it is an optimal area to study the ecological effects of urbanization and ecologization caused by land use changes in a karst region.

### 2.2. Data Source and Data Processing

**Figure 2:**
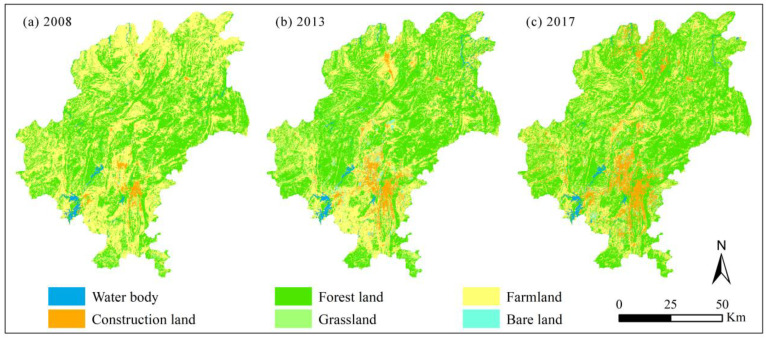
Map of land use distribution in Guiyang City in 2008 (**a**), 2013 (**b**), and 2017 (**c**).

In this study, the main sources of data used for EH assessment were Landsat-5 Thematic Mapper (TM) images for 2008 and Landsat-8 OLI images for 2013 and 2017. Remote satellite images were collected from the International Scientific and Technical Data website of the Chinese Academy of Sciences (http://www.gscloud.cn/(accessed on 10 December 2021)) [40]. The cloud content of all of the images was less than 5%, and the coordinate system of all images was uniformly resampled to the WGS_1984_UTM_Zone_48N projection coordinate system with a spatial resolution of 30 m × 30 m. Based on the ENVI 5.3 software platform (ITT Visual Information Solutions, Herndon, VA, USA), all of the images for each period were pre-processed by radiometric calibration, atmospheric correction, geometric correction, image enhancement, image mosaic and image subset, etc. Furthermore, according to China’s land use classification standard (GB/T 21010–2017), the land use types were classified into 6 categories (farmland, forestland, grassland, construction land, water body, and bare land) using the support vector machine supervision classification method based on the ENVI 5.3 software platform (Figure 2). The classification accuracy of each land use type and the overall classification accuracy of each period were more than 90%, with the kappa coefficient greater than 0.85.

### 2.3. Assessment Framework of Ecosystem Health

The vigor–organization–resilience model (VOR), proposed by Constanza [13], is a widely used model to assess ecosystem health (EH). In the VOR model, EH is measured through three aspects: vigor indicates ecosystem metabolism and primary productivity; organization represents ecosystem diversity, connectivity, and interactions; and resilience measures the capability to rebound from perturbations, and resilience of maintaining ecosystem structure and function when there is interference [1,13,23].

#### 2.3.1. Ecosystem Vigor (V)

As the normalized difference vegetation index (*NDVI*) is significantly positive correlated to primary productivity, it has generally been used to measure the ecosystem vigor [1,11]. Based on the band ratio method, *NDVI* is defined as the ratio of the difference between the near infrared band (*NIR*) and the visible red band (*R*) [1]. *NIR* and *R* are the fourth and third bands for Landsat TM images, and the fifth and fourth bands for Landsat OLI images. The value of *NDVI* ranges from 0 to 1, and a value close to 1 indicates relatively higher productivity (vigor). The formula of *NDVI* is as following:

(1) $$NDVI=\frac{NIR-R}{NIR+R}$$

#### 2.3.2. Ecosystem Organization (O)

Ecosystem organization refers the stability of ecosystem structure, which is mainly reflected in the diversity of the natural landscape and the impact of human activities, and can be quantitatively measured by landscape pattern indices, such as landscape heterogeneity, connectivity, and shape [1,11,41,42]. In the present study, the Shannon diversity index (SHDI) and Shannon evenness index (SHEI) were selected to measure landscape heterogeneity (LH). The cohension index (COHESION), contagion index (CONTAG), and landscape division index (DIVISION) were used to calculate landscape connectivity (LC). Landscape shape (LS) was assessed using the area-weighted mean fractal index (AWMFDI) and mean perimeter-area ratio index (MNPARA) [2,7]. The landscape pattern indices were calculated by using a moving window method based on the land use datasets of different periods in Fragstats 4.2 software. According to the relevant literature on ecosystem health assessment [11,17,18], the weight of each index and sub-index was assigned using the analytic hierarchy process (AHP). The weight of each index is shown in Table 1, among which consistency ratio (CR) was 0.0019 (<0.10). The formula of ecosystem organization (O) was set as follows:

O = 0.7153 × LH + 0.1870 × LC + 0.0977 × LS = (0.4769 × SHDI + 0.2384 × SHEI) + (0.0255 × COHESION + 0.1169 × CONTAG + 0.0446 × DIVISION) + (0.0814 × AWMFDI + 0.0163 × MNPARA)(2)

#### 2.3.3. Ecosystem Resilience (R)

Ecosystem resilience, which can also be referred to as ecosystem elasticity, refers to ability of ecosystem structures and behavioral patterns to rebound to the initial stage following human or natural disturbances [1]. A healthy ecosystem has enough resilience to withstand various forms of interference [7,10]. Ecosystem resilience can be characterized by the resistance and resilience to external disturbances [1,11]. Based on previous studies [1,7], resistance and resilience were given a weight of 0.6 and 0.4, respectively (Table 2). Ecosystem resilience was calculated using the following equation:

R = 0.6 × Resis + 0.4 × Resil(3)

where, ER refers to the ecosystem resilience, ‘Resis’ and ‘Resil’ refer to the resistance coefficient and resilience coefficient, respectively.

#### 2.3.4. Normalization and Classification of Ecosystem Health (EH)

**Figure 3:**
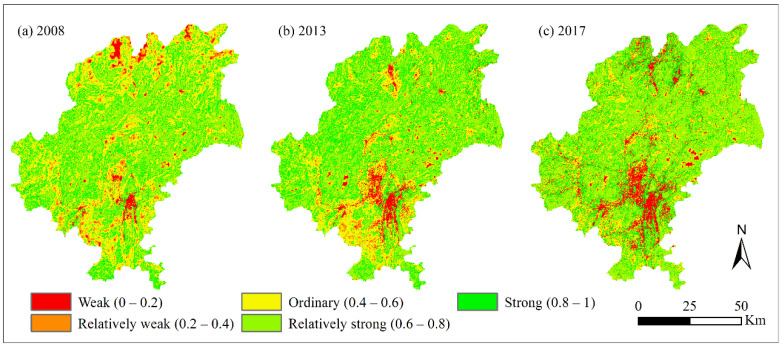
Distribution map of different ecosystem health classes in 2008 (**a**), 2013 (**b**) and 2017 (**c**).

In this study, the EH of Guiyang City was assessed according to Costanza’s definition [13] and Yan’s study [23], which applied the VOR framework. The formula of the VOR model is as follows:

EH = V × O × R(4)

where EH is the ecosystem health assessment score, and V, O, and R represent ecosystem vigor, organization, and resilience, respectively. EH indices at different periods were normalized to be comparable by using maximum difference normalization method with the range of [0,1]. Then, EH was divided into 5 levels with the intervals of 0.2: strong, 0.8 to 1; relatively strong, 0.6 to 0.8; ordinary, 0.4 to 0.6; relatively weak, 0.2 to 0.4; and weak, 0 to 0.2 (Figure 3).

### 2.4. Comprehensive Analysis of Landscape Patterns and Changes in EH

#### 2.4.1. Impact of Land Use Landscape Changes on EH

**Figure 4:**
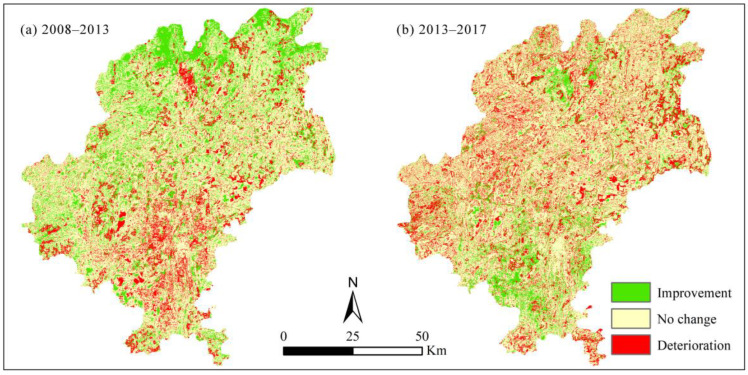
Ecosystem health changes in Guiyang City in 2008–2013 (**a**) and 2013–2017 (**b**). ‘Improvement’, the improved areas of ecosystem health; ‘No change’, the no change areas of ecosystem health; ‘Deterioration’, the deteriorated areas of ecosystem health.

To reveal the impact of land use landscape changes on EH, we divided EH and land use change into 2 phases: 2008–2013 and 2013–2017. First, the raster calculator tool of the ArcGIS 10.8 software platform (Environmental Systems Research Institute, Inc., Redlands, CA, USA) was used to extract the spatial transfer maps of different EH levels and determine the improved areas and deteriorated areas in 2008–2013 and 2013–2017, respectively (Figure 4); the tool was also used to extract the land use spatial transfer maps of those two periods [43]. Then, using the raster spatial overlay analysis method provided by ArcGIS 10.8 software, EH and land use spatial transfer maps in the different periods were overlaid to calculate the area of different land use transfer directions in EH improvement areas and deterioration areas.

#### 2.4.2. Impact of Landscape Composition and Configuration on EH

**Figure 5:**
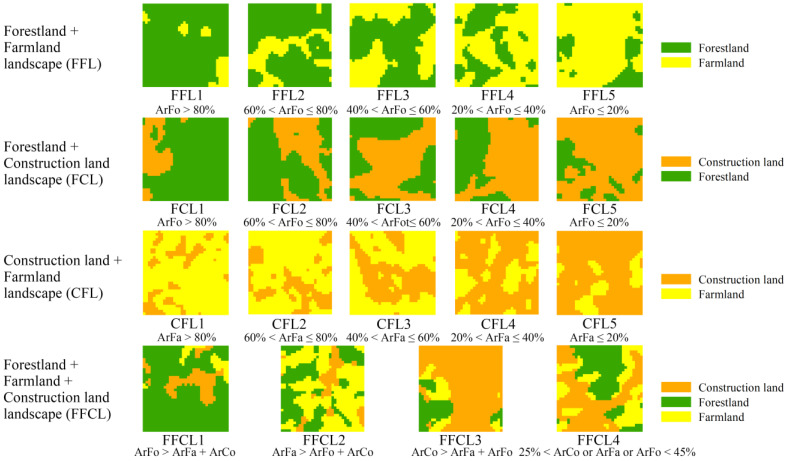
Schematic diagram of different landscape composition types. ArFo, ArFa, and ArCo indicate the area ratio of forestland, farmland, and construction land in 1 km × 1 km grid of different landscape types, respectively.

To explore the impact and relationship of the landscape matrix, composition, and spatial configuration on EH, the landscape type and ecological health index datasets of the study area in 2017 were divided into 1 km × 1 km grids using the Create Fishnet tool in ArcGIS 10.8. Based on the 1 km × 1 km grid scale, taking the 3 typical dominant landscape types (forestland, farmland, and construction land) as representatives, 4 typical landscape composition types (forestland + farmland landscape (FFL), forestland + construction land landscape (FCL), construction land + farmland landscape (CFL), and forestland + farmland + construction land mixed landscape (FFCL)) were selected, with a total sample number of 857. Then, according to the area ratio of landscape composition in each landscape type, FFL, FCL, and CFL were further divided into five landscape subtypes and FFCL was divided into four subtypes (Figure 5).

Dunnett’s T3 ANOVA analysis was conducted in order to analyze the differences in ecological health among the different landscape composition types and sub types. In addition, eight landscape metrics at the landscape level (SHEI, SHDI, SPLIT, LSI, DIVISION, AWFRAC, LPI, and CONTAG) were selected to represent the landscape spatial configuration, and Spearman correlation analysis was conducted to analyze the relationship between the average ecological health index of different landscape composition types and landscape spatial configuration metrics. The landscape metrics for each gird were calculated using Fragstats 4.2 software.

## 3. Results

### 3.1. Land Use Dynamics from 2008 to 2017

The land use pattern of Guiyang City notably changed from 2008 to 2017 (Figure 2). Farmland and forestland were the main land use types, with their area accounting for more than 86% of the total area. Due to the coupled influence of rapid urbanization and substantial ecologization, the area of farmland continuously decreased, whereas forestland became the dominant land use types since 2013, accounting for 51.86% and 56.13% in 2013 and 2017, respectively. Meanwhile, the area of construction land significantly increased by 167.27% and 91.12% in 2008–2013 and 2013–2017, respectively (Table 3).

### 3.2. Changes in EH from 2008 to 2017

The EH of the study area was at a relatively strong level, with an average EH value of 0.63, 0.65, and 0.62 in 2008, 2013, and 2017, respectively. During the study period, EH was mainly classified as ordinary, relatively strong, and strong, with these 3 classes accounting for more than 93% of the total study area. From 2008 to 2013, areas with relatively strong and strong levels increased, whereas the relatively weak level significantly decreased, and EH was slightly improved in this period. From 2013 to 2017, the relatively weak class was further reduced, only accounting for 0.05% of the total study area, and the area of ordinary and strong classes also decreased. Meanwhile, the area of weak and relatively strong classes increased to varying degrees (Table 4). On the whole, the EH changes show the coupled characteristics of deterioration and improvement in 2008–2017. As for the spatial distribution of EH values (Figure 3), lower values were mainly concentrated in and around the built-up area located in the south-central part of the study area with strong human interference, while higher values were mainly distributed in the ecological land region (forestland, grassland).

### 3.3. Relationship between Landscape Types Change and Ecosystem Health

**Figure 6:**
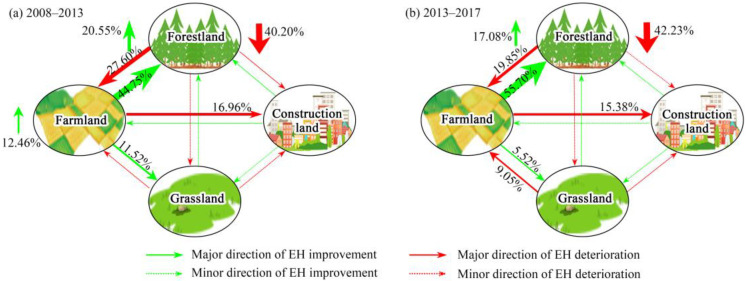
The contribution ratio of different land use transfer directions on ecosystem health (EH) improvement or deterioration in 2008–2013 (**a**) and 2013–2017 (**b**).

Figure 4 and Figure 6 show the EH changes and correspond to the transfer of landscape types between 2008 and 2017. The area of deteriorated EH was 1269.33 and 1710.43 km^2^ in 2008–2013 and 2013–2017, respectively. The main contributors were forest land degradation (40.20 and 42.23%), transfer of forest land to farmland (27.60 and 19.85%), and transfer of forest land to construction land (16.96 and 15.38%) (Figure 4a and Figure 6). By contrast, the area of improved EH was 1815.31 and 1107.92 km^2^ in 2008–2013 and 2013–2017, respectively, and the transfer of farmland to forestland, forestland improvement, and transfer of farmland to grassland driven by ecological restoration measures were the main contributors accounting for 44.75, 20.55, and 11.52% in 2008–2013 and 55.70, 17.08, and 5.52% in 2013–2017, respectively (Figure 4b and Figure 6). This indicates that rapid expansion of urbanization or agriculturalization could cause a decline in EH, whereas ecological construction and restoration could be effective measures to improve EH.

### 3.4. Influence of Landscape Composition and Configuration on Ecosystem Health

**Figure 7:**
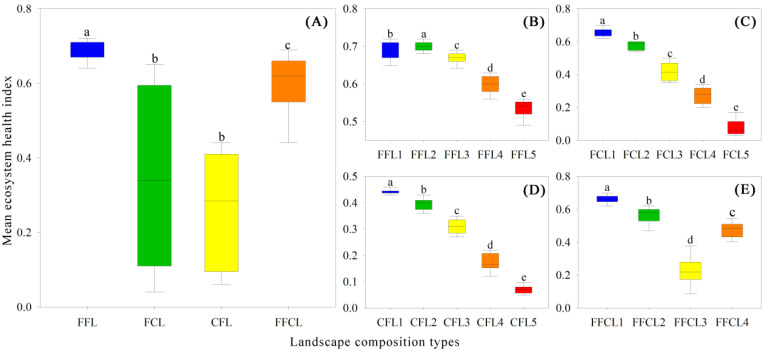
Ecosystem health differences between landscape composition types. (**A**) differences between four landscape types; (**B**) difference between five subtypes of forestland + farmland landscape; (**C**) difference between five subtypes of forestland + construction land landscape; (**D**) difference between five subtypes of construction land + farmland landscape; (**E**) difference between four subtypes of forestland + farmland + construction land landscape. FFL, forestland + farmland landscape; FCL, forestland + construction land landscape; CFL, construction land + farmland landscape; FFCL, forestland + farmland + construction land landscape. Same letters indicate no significant differences between landscape types, and different letters indicate significant differences types based on Dunnett’s T3 ANOVA analysis (*p* < 0.05).

Figure 7 shows the difference in EH of four landscape composition types (FFL, FCL, CFL, and FFCL). It was found that the landscape composition had a significant effect on EH (*p* < 0.05). The average EH of the FFL type was significantly higher than that of FCL, CFL, and FFCL (*p* < 0.05), and there was not significant difference between FCL and CFL (*p* > 0.05), with the average EH value of these 2 types below 0.4 (Figure 7A). In addition, different proportions of landscape components in the same landscape type have significantly different effects on EH (*p* < 0.05). With a reduced proportion of forestland or farmland, the average EH of FFL, FCL, and CFL significantly decreased. The average EH of FFL was at the ordinary or relatively weak level when the proportion of forestland was less than 40% (Figure 7B). The average EH of FCL and CFL was at the relatively weak or weak levels when the proportion of forestland in FCL and farmland in CFL was less than 60% and 80%, respectively (Figure 7C,D). As for the mixed FFCL, when the proportion of construction land was greater than the total area of forestland and farmland, the average EH of FFCL (FFCL3) was closed to the weak level, whereas the EH of other FFCL types (FFCL1, FFCL2, and FFCL4) was significantly higher than that of FFCL1 (*p* < 0.05), with the average EH of ordinary, relatively strong, and strong levels (Figure 7E).

**Figure 8:**
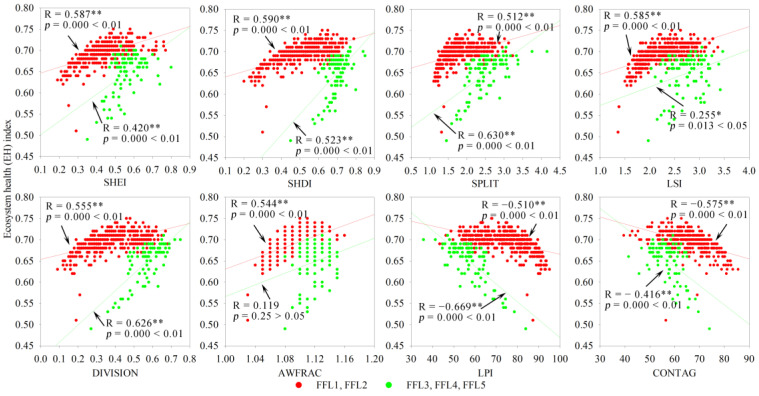
Correlation of landscape composition and configuration with the ecosystem health in forestland + farmland landscape. FFL, forestland + farmland landscape; SHEI, Shannon’s evenness index; SHDI, Shannon’s diversity index; SPLIT, splitting index; LSI, landscape shape index; DIVISION, landscape division index; AWFRAC, area-weighted mean fractal dimension index; LPI, largest patch index; CONTAG, contagion index. * *p*-values at 5% level; ** *p*-value at 1% level.

**Figure 9:**
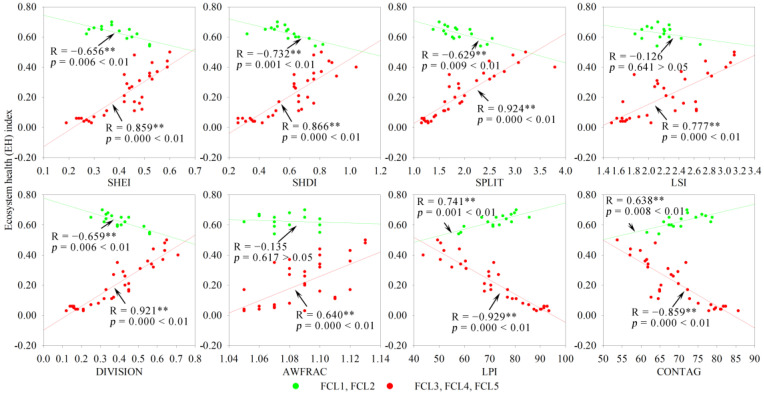
Correlation of landscape composition and configuration with ecosystem health in forestland + construction land landscape. FCL, forestland + construction land landscape; SHEI, Shannon’s evenness index; SHDI, Shannon’s diversity index; SPLIT, splitting index; LSI, landscape shape index; DIVISION, landscape division index; AWFRAC, area-weighted mean fractal dimension index; LPI, largest patch index; CONTAG, contagion index. ** *p*-value at 1% level.

**Figure 10:**
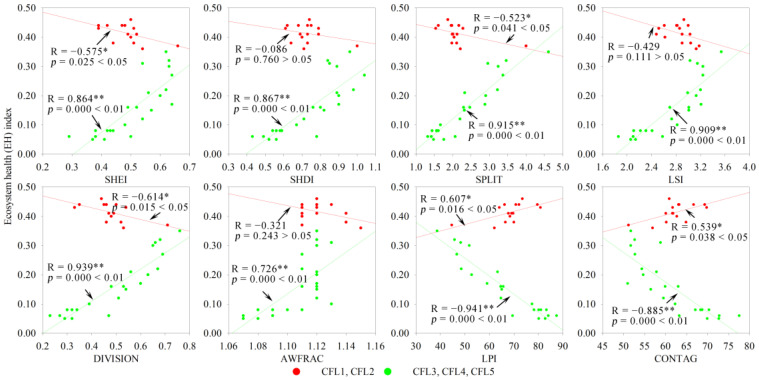
Correlation of landscape composition and configuration with ecosystem health in construction land + farmland landscape. CFL, construction land + farmland landscape; SHEI, Shannon’s evenness index; SHDI, Shannon’s diversity index; SPLIT, splitting index; LSI, landscape shape index; DIVISION, landscape division index; AWFRAC, area-weighted mean fractal dimension index; LPI, largest patch index; CONTAG, contagion index. * *p*-values at 5% level; ** *p*-value at 1% level.

**Figure 11:**
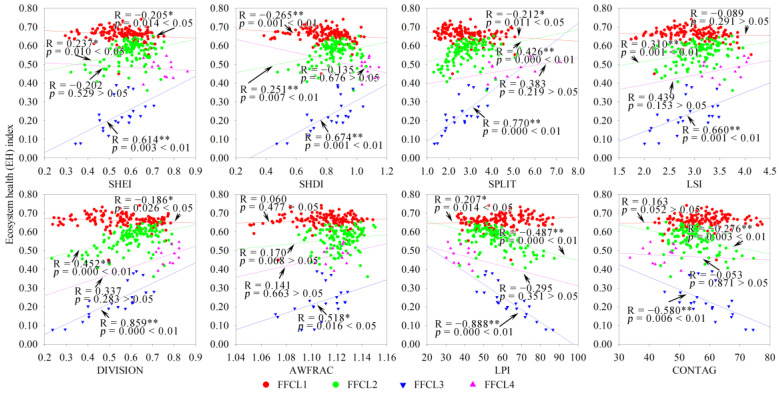
Correlation of landscape composition and configuration with ecosystem health in forestland + farmland + construction land mixed landscape. FFCL, forestland + farmland + construction land mixed landscape; SHEI, Shannon’s evenness index; SHDI, Shannon’s diversity index; SPLIT, splitting index; LSI, landscape shape index; DIVISION, landscape division index; AWFRAC, area-weighted mean fractal dimension index; LPI, largest patch index; CONTAG, contagion index. * *p*-values at 5% level; ** *p*-value at 1% level.

Based on the 1 × 1 km grid scale, the landscape configuration was shown to have a significant or highly significant negative or positive correlation with the EH; however, the correlations differed in different landscape composition types and subtypes. For FFL, FCL and CFL dominated by construction land (FCL3, FCL4, FCL5, CFL3, CFL4, and CFL5), and FFCL dominated by farmland (FFCL2), SHEI, SHDI, LSI, DIVISION, and AWFRAC indices had a significant (*p* < 0.05) or extremely significant (*p* < 0.01) positive correlation with EH, whereas the LPI and CONTAG indices had a significant or extremely significant negative correlation with EH (Figure 8, Figure 9, Figure 10 and Figure 11). The opposite phenomenon was found for FCL and FFCL dominated by forestland (FCL1, FCL2, and FFCL1) and CFL dominated by farmland (CFL1 and CFL2); however, the correlation between landscape pattern indices and EH also displayed a significant or extremely significant relationship (Figure 9, Figure 10 and Figure 11). In addition, for FFCL with similar proportions of different landscape components (FFCL4), EH had a negative correlation with SHEI, SHDI, LPI, and CONTAG indices and a positive correlation with SPLIT, LSI, DIVISION, and AWFRAC; however, the correlation between EH and these indices in FFCL was not significant (*p* > 0.05) (Figure 11).

## 4. Discussion

### 4.1. Response of Ecosystem Health to Land Use Changes

Previous studies have shown that land use changes can lead to distinct environmental and socioeconomic changes, which in turn affect ecosystem services and ecological health [2,44]. Land use change, especially urbanization expansion, leads to large-scale environmental degradation and is the main driver of ecological health degradation [11,19]. The fragile ecological system of karst mountainous areas in China has weak self-recovery ability of the landscape after disturbance, with the high potential risk to ecological health [31,45]. With economic growth, increased population, and social development in recent years, urbanization expansion has become the main trend and direction of land use change in karst mountainous areas, which poses a serious threat to ecological health [37,38].

In this study, it was found that areas with poor ecosystem health in Guiyang City were mainly distributed in and around built-up areas with a concentration of construction land. Areas with degraded ecosystem health were also mainly concentrated in the urbanization expansion region (Figure 2 and Figure 3). This finding is similar to the results of studies in the Golden Triangle of Southern Fujian Province, China [19], and in the Kolkata Metropolitan Area in India [11]. Therefore, this demonstrates that changes in the land use structure by urbanization expansion is the main reason for ecosystem health degradation. This also agrees with findings in other studies [2,35,46]. On the other hand, large-scale ecological restoration projects (such as the Natural Forest Protection Project, the Grain to Green Program, the Public Welfare Forest Protection, and the Karst Rocky Desertification Restoration Project) have been implemented to protect the fragile ecological environment, improve the ecological quality, and maintain the ecosystem services in karst mountainous region in China [18]. More than USD 19 billion has been invested in ecological restoration projects since the end of the 1990s, and most of the farmlands on sloped hills has been abandoned and is currently covered by shrubs, tree plantations, or secondary and man-made forests [18,31,47]. In this way, ecological restoration measures have produced positive ecological effects, with significant improvements in vegetation coverage (ecosystem vigor) and ecosystem resilience and services [48,49].

Due to the high ecological vigor and resilience of the natural ecosystem dominated by forestland, the areas with relatively strong and strong levels of ecosystem health were distributed in the forestland and ecological restoration areas (Figure 4 and Figure 6), which is supported by previous studies [18]. In addition, with regards to the distribution and change in ecosystem health of Guiyang City in 2008–2017, the overall ecosystem health of the study area was relatively strong, with the average ecosystem health index greater than 0.6 (Figure 3). Ecological restoration measures led to optimization of the land use structure and retarded the deterioration of ecosystem health caused by expanding urbanization. However, with the intensification of rapid urban expansion, the trend of ecosystem health degradation was still severe, especially in and around the built-up area (Figure 2 and Figure 3). Therefore, it is necessary to further strengthen ecological restoration and protection measures, conserve natural ecosystems, optimize the quantitative structure and spatial patterns of land use in urbanization areas, and limit the sprawl of construction land, and aim for sustainable ecological development [2].

### 4.2. Relationship between Landscape Structure and Ecosystem Health

The landscape structure, comprising landscape composition and configuration, is an indicator reflecting the spatial patterns of the ecosystem and the connectivity between different landscape elements [50,51,52]. Landscape composition and configuration not only directly affect energy and material flows, but also impact particular ecosystem services and regional ecosystem functions [53,54]. Thus, incorporating landscape structure into the assessment of ecosystem health is considered a suitable for assessing comprehensive ecosystem functions on the macro scale [2]. In addition, the impact of landscape structure on ecosystem functions and services and human well-being is one of the core issues in landscape sustainability science [55]. Specific landscape structures composed by different landscape matrices can generate distinct ecological effects.

In this study, the four landscape composition types (FFL, FCL, CFL, and FFCL) represent the results of different forms and degrees of human interference in the landscape and can reflect the different stages of landscape evolution. It indicates that the overall ecosystem health of the landscape type with a natural or semi-natural ecosystem (FFL) was significantly better than that of landscape types with an artificial ecosystem (FCL and CFL). Different proportions of compositions in the same landscape types have various effects on ecosystem health (Figure 7). For example, in the natural and semi-natural landscape type (FFL), when the area ratio of the natural ecosystem type (forestland) was not less than 40% at the 1 km × 1 km grid scale, ecosystem health could be maintained at a relatively good level. While this law varied in other landscape types, the area ratio of forestland in FCL and farmland in CFL should be greater than 60% and 80%, respectively, to effectively maintain ecosystem health at a relatively good level (Figure 7C,D). This finding provides a valuable reference basis (threshold) for the optimization and planning of land use spatial patterns and environmental management in karst mountainous regions.

Changes in landscape composition metrics play a direct role in landscape configuration, and then jointly affect ecological functions and processes, leading to changes in ecosystem services and health [51,54,56]. However, the interactions between landscape pattern changes and ecological processes are not unidirectional, and the complexity of the interaction between landscape structure and function determines the diverse impacts of landscape configuration on ecosystem health [2]. The relationship between the configurations of landscape matrix types and ecosystem health usually shows a significant difference. For instance, in this study, it was found that ecosystem health in the landscape types dominated by construction land (FCL3, FCL4, FCL5, CFL3, CFL4, and CFL5) was significantly negatively correlated with LPI and CONTAG and significantly positively correlated with SHEI, SHDI, SPLIT, LSI, DIVISION, and AWFRAC, in contrast to landscape types dominated by forestland (FCL1, FCL2, and FFCL1) or farmland (CFL1 and CFL2) (Figure 8, Figure 9, Figure 10 and Figure 11). LPI, as a typical landscape composition metric, reflects the ability of landscape patches to resist fragmentation, and can explain landscape homogeneity, whereas CONTAG represents landscape clumpiness and aggregation. A higher CONTAG value indicates that the landscape patch types are clumped and have lower spatial diversity [50,53]. As for the landscape types dominated by artificial ecosystems (FCL3, FCL4, FCL5, CFL3, CFL4, and CFL5), higher LPI and CONTAG values clearly suggest the dominance of construction land in the landscape with a concentrated distribution. This can lead to obstacles to for material circulation and energy flow in the landscape, reduce the ecological connectivity within the landscape, and then result in the degradation of ecosystem health. Accordingly, strengthening landscape uniformity, connectivity, and diversity, increasing the complexity of landscape patch shapes, and promoting the internal ecological flow of the landscape through the edge effect might be an effective strategy to maintaining ecosystem health. However, for landscape types dominated by a natural or semi-natural ecosystem (FCL1, FCL2, FFCL1, CFL1, and CFL2), maintaining the integrity of natural ecosystems and habitats, reducing landscape fragmentation and complexity, and promoting the ecological function and process of landscape “sources” could be conducive to improving ecosystem health. Therefore, we considered that in and around built-up areas with a high degree of urbanization and rapid urban expansion, FCL with more than 60% of forestland and FCL with good natural ecosystem integrity and strong landscape connectivity are ideal landscapes for maintaining and improving ecosystem health in karst mountainous cities. Whereas in rural or peri-urban areas, aside from limiting the rapid expansion of urbanization and strengthening ecological restoration, improving the diversity, connectivity, and complexity of landscape patterns might be a reasonable measure for land use landscape planning and environmental management policymaking in the future.

### 4.3. Limitations and Future Work

Pattern or heterogeneity is the cornerstone concept and essential attribute of landscape ecology [57]. Diversity and heterogeneity of the landscape lead to the complexity of landscape ecological functions, processes, and services at different scales and in different regions. Undoubtedly, ecosystem health is a complex concept, and its assessment has been conducted at the ecosystem, landscape, regional, and global scales [7]. On the landscape scale, research on the relationship between specific landscapes caused by land use changes and ecosystem health provides an integrated view for ecosystem health assessment and environmental management [1,7,58]. This study found that there is indeed an inseparable relationship between landscape structure and ecosystem health, but it is not simply a positive or negative correlation in different landscape matrices, compositions, and configurations. The impact mechanism of various landscape structures on ecosystem health is still difficult to clarify. Thus, quantitatively describing the relationship between landscape structure and ecosystem health, exploring the response of regional ecosystem health to different landscape structures and the influencing factors, and revealing the interaction mechanism between landscape structures and ecosystem health remains to be analyzed more deeply in future research. Moreover, more attention should be paid to optimizing land use patterns and adopting effective environmental management measures at the landscape scale in order to maintain regional ecosystem health and achieve sustainable development of human society in the future.

## 5. Conclusions

We conclude the following: (1) the land use pattern in Guiyang City from 2008 to 2017 was dramatically changed. Rapid urbanization and ecologization were the major trends of land use changes. Forestland was the predominant land use type in 2017. (2) Overall ecosystem health was relatively strong. The increase in forestland area had a positive effect on EH, whereas the interference of human activities, especially urban and agricultural expansion, posed a serious threat to EH at this stage. (3) The EH of the natural and semi-natural landscape type was superior to that of other landscape types. The EH of landscape types dominated by construction land and forestland + farmland was significantly negatively correlated with LPI and CONTAG, and significantly positively correlated with SHEI, SHDI, LSI, SPLIT, DIVISION, and AWFRAC. However, it was opposite for landscape types dominated by forestland or farmland. The findings of this study on the response of EH to different land use changes and landscape structures can provide guidance and reference for landscape pattern planning and environmental management policy formulation at different development stages in the karst mountainous regions.

## Figures and Tables

**Table 1:** Weights of criterion and sub-index layers of ecosystem organization using AHP.

Criterion Layer	LH		LC			LS	
LH	1		1/4			1/7	
LC	4		1			1/2	
LS	7		2			1	
Wic	0.7153		0.187			0.0977	
CR	0.0019						
sub-index layer	SHDI	SHEI	COHESION	CONTAG	DIVISION	MNPARA	AWMFDI
SHDI	1	1/2					
SHEI	2	1					
COHESION			1	4	2		
CONTAG			1/4	1	1/3		
DIVISION			1/2	3	1		
MNPARA						1	5
AWMFDI						1/5	1
Wis	0.6667	0.3333	0.1365	0.6250	0.2385	0.1667	0.8333
CR	0.0000		0.0176			0.0000	
Wii	0.4769	0.2384	0.0255	0.1169	0.0446	0.0814	0.0163

Note: LH, landscape heterogeneity; LC, landscape connectivity; LS, landscape shape; Wic, weight of criterion layers; Wis, weight of sub-index layers; Wii, weight of each ecosystem organization index; CR, consistency ratio; SHDI, Shannon diversity index; SHEI, Shannon evenness index; COHESION, cohension index; CONTAG, contagion index; DIVISION, landscape division index; MNPARA, mean perimeter-area ratio index; AWMFDI, area-weighted mean fractal index.

**Table 2:** The ecosystem resilience coefficient of each landscape type in Guiyang City.

Landscape Type	Forestland	Grassland	Farmland	Construction Land	Water Body	Bare Land
Resis	1.0	0.6	0.5	0.3	0.8	0.2
Resil	0.6	0.8	0.3	0.2	0.7	1.0
R	0.84	0.68	0.42	0.26	0.76	0.52

Note: ‘Resis’ and ‘Resil’ refer to the resistance coefficient and resilience coefficient, respectively. ‘R’ refers to ecosystem resilience.

**Table 3:** The area and ratio of different land use types in 2008, 2013 and 2017.

Land Use Types	2008		2013		2017		Change in Land Use (%)		
	Area (km^2^)	Ratio (%)	Area (km^2^)	Ratio (%)	Area (km^2^)	Ratio (%)	2008–2013	2013–2017	2008–2017
Farmland	3703.08	48.51	2763.64	36.20	2297.61	30.10	−25.37	−16.86	−37.95
Forestland	3426.42	44.88	3959.30	51.86	4285.26	56.13	15.55	8.23	25.07
Grassland	260.95	3.42	290.53	3.81	130.39	1.71	11.34	−55.12	−50.03
Construction land	137.83	1.81	368.37	4.83	704.04	9.22	167.27	91.12	410.82
Water body	92.57	1.21	155.61	2.04	97.17	1.27	68.10	−37.55	4.97
Bare land	13.48	0.18	96.87	1.27	119.85	1.57	618.69	23.72	789.18

**Table 4:** Area and ratio of different ecosystem health classes in 2008, 2013, and 2017.

Ecosystem Health Level	2008		2013		2017	
	Area (km^2^)	Ratio (%)	Area (km^2^)	Ratio (%)	Area (km^2^)	Ratio (%)
Weak	240.14	3.15	411.72	5.39	750.17	9.83
Relatively weak	250.49	3.28	52.73	0.69	3.61	0.05
Ordinary	3467.53	45.42	2901.20	38.01	2486.02	32.58
Relatively strong	1558.71	20.42	1739.67	22.79	2236.21	29.30
Strong	2117.45	27.74	2527.38	33.11	2155.52	28.24

## Data Availability

Not applicable.
